# Effects and Mechanisms of the Xianhecao-Huanglian Drug Pair on Autophagy-Mediated Intervention in Acute Inflammatory Bowel Disease via the JAK2/STAT3 Pathway

**DOI:** 10.1186/s12575-024-00242-5

**Published:** 2024-08-26

**Authors:** Yaping He, Xinling Shen, Haiyan Peng

**Affiliations:** 1 The Affiliated Hospital of Nanjing University of Chinese Medicine, Nanjing, 210023 Jiangsu China; 2 The First Clinical College of Nanjing University of Chinese Medicine, Nanjing, 210029 Jiangsu China

**Keywords:** Inflammatory bowel disease (IBD), Xianhecao-Huanglian drug pair, Autophagy, JAK2/STAT3 signaling pathway

## Abstract

To explore the effects and mechanisms of the Xianhecao-Huanglian drug pair on autophagy-mediated intervention in acute inflammatory bowel disease (IBD) via the JAK2/STAT3 pathway. The study examined the underlying mechanisms of action of Xianhecao (APL) and Huanglian (CR) using a mouse model of dextran sodium sulfate (DSS)-induced acute inflammatory bowel disease (IBD) and in an in vitro model of IBD induced by lipopolysaccharide (LPS). The assessment of the therapeutic efficacy of the Xianhecao-Huanglian drug combination in a mouse model of IBD caused by DSS included the following parameters: Assessment of weight loss or gain. Measurement of the disease activity index (DAI). Assessment of histological damage. Determination of organ index. Measurement of colon length. Ascertain the levels of inflammatory cytokines in the intestinal tissues and serum of mice. Immunohistochemistry (IHC) for the measurement of tight junction protein concentrations in the colon mucosa, including ZO-1, claudin-1, and occludin. Measurement of mucin levels, specifically Mucin 2 (Muc2). Hematoxylin and eosin (HE) staining for the observation of histopathological alterations in colonic tissues. Examining the effect on goblet cells using periodic acid-Schiff (PAS) labeling. Application of Western blot and immunofluorescence techniques for the detection of autophagy-related markers in colonic tissues and proteins associated with the JAK2/STAT3 pathway. A cell inflammation model of IBD was induced through LPS stimulation, and a serum containing the Xianhecao-Huanglian drug pair (referred to as ACHP-DS) was formulated. Cell viability, anti-proinflammatory cytokines, tight junction proteins, mucins, autophagy-related markers, and the JAK2/STAT3 signaling pathway were assessed. The Xianhecao-Huanglian drug pair significantly ameliorated the symptoms and survival quality of acute IBD mice, reducing the disease activity index score, raising MUC2 secretion and tight junction protein expression to improve the integrity of the intestinal barrier, and preserving goblet cell function; thus, protecting the intestines. It effectively restrained triggering the signaling pathway that involves JAK2 and STAT3, leading to the suppression of inflammation and amelioration of colonic inflammation damage. Additionally, it induced autophagy in mouse colonic tissues.The in vitro experiments demonstrated that the Xianhecao-Huanglian drug combination enhanced the viability of LOVO and NCM460 cells when exposed to LPS stimulation. Furthermore, it suppressed the production of inflammatory cytokines such as IL-6, IL-1β, as well as TNF-α, whilst increasing the production of IL-10, ZO-1, along with MUC2. These effects collectively led to the alleviation of inflammation and the restoration of mucosal integrity. The results were consistent with what was shown in the in vivo trial. Moreover, the medication demonstrated effectiveness in reducing JAK2 along with STAT3 phosphorylation levels in the LPS-induced inflammatory model of IBD cells. The intervention with either the Xianhecao-Huanglian drug combination-containing serum or the JAK2/STAT3 pathway inhibitor AG490 reversed the pro-inflammatory effects and increased autophagy levels in the LPS-stimulated cells. The Xianhecao-Huanglian drug combination modulates the JAK2/STAT3 pathway, leading to the induction of autophagy, which serves as an intervention for IBD.

## Background

A complex inflammatory illness affecting the gastrointestinal tract, inflammatory bowel disease (IBD) is characterized by inflammation of the colonic as well as rectum mucosa. Illnesses such as ulcerative colitis (UC) as well as Crohn’s disease (CD) fall within this category. Clinical manifestations include recurring mucus-laden bloody stools, diarrhea, and chronic wasting, often leading to severe local or systemic complications [1]. IBD is frequently linked with persistent intestinal inflammation and tissue damage, and this sustained inflammation in the gastrointestinal tract significantly accelerates the process of “inflammation-carcinogenesis,” elevating the risk of colorectal cancer (CRC) in individuals with IBD. The incidence and prevalence of IBD are on the rise at a concerning rate in both developed and developing countries. It is widely believed to be a multifactorial ailment influenced by genetic and environmental factors [2]. Research has established a close correlation between acute IBD and colorectal cancer, and the genesis and progression of this condition are associated with genetic or epigenetic alterations in oncogenes and tumor suppressor genes in an inflammatory environment. In contrast to patients with sporadic colorectal cancer (S-CRC), individuals with colitis-associated colorectal cancer (CA-CRC) tend to experience cancer earlier in life, exhibit a higher degree of malignancy, and frequently experience progression due to poorly defined flat dysplasia within the inflammatory setting. Many patients can effectively mitigate the risk of cancer development by undergoing treatment and managing inflammation to minimize ongoing damage and erosion of the colorectal mucosa. Early surveillance and intervention in IBD perform an essential function in the detection, evaluation, and management of colorectal cancer in its early stages. Given the recurrent and often challenging nature of IBD, as well as its various complications, surgical resection may offer a potential cure in certain instances [3]. However, this procedure is associated with considerable medical expenses, and most young patients exhibit hesitance toward a lifelong ileostomy. IBD is a chronic ailment, and the prevailing medical interventions include five primary drug categories: aminosalicylates, glucocorticoids, immunomodulators, antibiotics, and biologics. Nonetheless, it is imperative to note that there remains no definitive cure for IBD. Owing to constraints associated with the prolonged utilization of anti-inflammatory or immunosuppressive medications, the focus has shifted toward exploring the potential of Traditional Chinese Medicine (TCM) for managing IBD.

Inflammatory bowel disease (IBD) is intricately linked to dysregulated immune responses within the gastrointestinal tract, where the JAK2/STAT3 pathway assumes a pivotal role. This pathway, involving Janus Kinase 2 (JAK2) and Signal Transducer and Activator of Transcription 3 (STAT3), orchestrates the inflammatory cascade, modulating various aspects of immune function and tissue homeostasis. In the context of IBD, aberrant activation of the JAK2/STAT3 pathway is a hallmark feature, driving sustained inflammation and tissue damage [4]. Upon stimulation by cytokines such as interleukin-6 (IL-6) and interleukin-23 (IL-23), JAK2 phosphorylates and activates STAT3, leading to its dimerization and translocation to the nucleus. Within the nucleus, activated STAT3 acts as a transcription factor, regulating the expression of genes involved in inflammation, cell proliferation, and survival. The importance of the JAK2/STAT3 pathway in IBD pathogenesis lies in its ability to perpetuate the inflammatory milieu within the gastrointestinal mucosa. Persistent activation of STAT3 promotes the production of pro-inflammatory cytokines, such as tumor necrosis factor-alpha (TNF-α), interleukin-1β (IL-1β), and interleukin-17 (IL-17), exacerbating tissue damage and disrupting epithelial barrier integrity [5]. Moreover, STAT3 activation within immune cells enhances their recruitment to the inflamed gut mucosa, further fueling the inflammatory response. Beyond its role in inflammation, the JAK2/STAT3 pathway influences key processes implicated in IBD progression, including epithelial cell turnover, mucosal healing, and the balance between regulatory and effector immune responses. Dysregulated STAT3 signaling has been implicated in the development of colitis-associated colorectal cancer (CA-CRC), a severe complication of long-standing IBD. STAT3 activation promotes epithelial cell proliferation and survival, contributing to the emergence of dysplastic lesions and malignant transformation. Targeting the JAK2/STAT3 pathway represents a promising therapeutic strategy for IBD management. Inhibition of JAK2 or STAT3 activity has been shown to ameliorate colitis severity and promote mucosal healing in preclinical models of IBD. Moreover, emerging biologic therapies targeting JAK2/STAT3 signaling have demonstrated efficacy in reducing disease activity and inducing clinical remission in patients with moderate to severe IBD [6, 7]. Autophagy assumes a crucial role in upholding normal cellular operations by disassembling proteins and cellular organelles, contributing to energy equilibrium, and functioning as a ubiquitous self-preservation mechanism within cells [8]. Among the genetic components implicated in the onset of IBD, variations in genes related to autophagy have been identified. Numerous clinical investigations and laboratory experiments have demonstrated the pivotal role of autophagy in preserving intestinal balance, regulating intestinal microorganisms, and coordinating appropriate immune responses in the intestines. Malfunctions in autophagy are known to significantly influence the progression of IBD, disrupting intestinal equilibrium, impacting changes in gut microbiota, reducing the removal of bacteria from cells, and worsening inflammation in the intestines [15]. Furthermore, research has indicated that the utilization of natural products that induce autophagy by IBD patients can alleviate the disease [10]. Noteworthy proteins implicated in the formation of autophagy include Beclin1, ATG12, and LC3A/B. ATG12, an ubiquitin-like protein, bears a resemblance to yeast proteins engaged in autophagy and forms an ATG12-ATG5 complex under the influence of ATG7 and ATG10 [11]. Beclin-1 primarily participates in the creation of autophagosomes, and its expression is positively correlated with autophagy levels [12]. After undergoing transformation, LC3-II binds to the membrane of the autophagosome. The presence of LC3 in autophagosomes and its conversion into LC3-II, detected as a migration pattern change, serve as indicators of autophagy. Assessing alterations in the LC3II/LC3I ratio is a widely employed technique for assessing autophagy levels. Additionally, P62 obstructs autophagy through interaction with LC3, infiltrating autophagosomes, and effectively undergoing degradation in autophagic lysosomes [13].

A substantial body of clinical studies has confirmed the renowned anti-inflammatory properties of TCM as they pertain to the management of IBD. TCM presents unique advantages in enhancing and improving the IBD patients’ quality of life, decreasing the likelihood of cancer progression and recurrence, and relieving symptoms. Drug pairs are a fundamental aspect of TCM compounds. Xianhecao (Agrimonia pilosa Ledeb. APL) and Huanglian (Coptis chinensis, C. chinensis) are two common heat-clearing and detoxifying herbs, often used as a drug pair used by Zhou Zhongying, Master of TCM, to the therapeutic management of gastrointestinal disorders. Xianhecao comprises various chemical components, including flavonoids, triterpenes, tannins, and phenolic compounds, and has a long history of utilization in treating enteritis [14]. Numerous proprietary Chinese medicine formulations containing Xianhecao as a major component, such as Compound Xianhecao Enteritis Tablets [14] and Fuan Granules, have been developed and widely promoted in clinical practice for treating gastrointestinal diseases. Furthermore, Xianhecao has a wide margin of safety for use. In contrast, Huanglian is exceptionally bitter and cold, with functions, including heat-clearing, dampness-drying, fire-purging, and detoxification. It comprises diverse ingredients, including alkaloids, lignans, flavonoids, and acidic components, which have demonstrated pharmacological effects such as anti-tumor, hypoglycemic, antimicrobial, anti-inflammatory, and digestive system improvement properties. Huanglian is effective in treating intestinal infections and gastrointestinal disorders and is frequently employed in the clinical treatment of IBD [16]. However, limited research has been conducted on the mechanism of action of the Xianhecao-Huanglian drug pair (APL-C. chinensis, ACHP) in combatting IBD.

The fundamental goal of this study was to investigate the effects and causes of the Xianhecao-Huanglian drug combination in the management of IBD. This investigation utilized a model of acute IBD in mice, being exposed to DSS, as well as an IBD inflammatory cell model provoked by lipopolysaccharide (LPS). The objective was to offer a contemporary molecular biology foundation for the intervention of IBD using the Xianhecao-Huanglian drug pair.

## Materials and Methods

### Antibodies and Reagents

The molecular weight of the DSS, which was between 36,000 and 50,000, was given by MP Biomedicals LLC. The main antibodies that were used were: Sources from which the following reagents were procured: Atg12 antibody (#4180T, Cell Signaling Technology), p62 antibody (#5114T, Cell Signaling Technology), Beclin-1 antibody (#3495T, Cell Signaling Technology), LC3A/B antibody (#12741T, Cell Signaling Technology), β-actin antibody (#4970T, Cell Signaling Technology), β-Tubulin antibody (#2128, Cell Signaling Technology), JAK2 antibody (ab108596, Abcam), Phosphorylated JAK2 antibody (P-JAK2, ab32101, Abcam), STAT3 antibody (ab68153, Abcam), Phosphorylated STAT3 antibody (P-STAT3, ab267373, Abcam), MUC2 antibody (ab272692, Abcam), Claudin-1 antibody (ab211737, Abcam), Occludin antibody (ab222691, Abcam), ZO-1 antibody (ab216880, Abcam), Elabscience (Wuhan) was also the source for the following ELISA kits: The following ELISA kits were purchased from Elabscience in Wuhan: Mouse TNF-α (E-EL-M3063), Mouse Interleukin-1β (IL-1β) (E-EL-M0037c), Mouse IL-6 (E-EL-M0044c), Mouse Interleukin-10 (IL-10) (E-EL-M0046c), Human TNF-α (E-EL-H0109c), Human Interleukin-1β (E-EL-H0149c), Human IL-6 (E-EL-H6156), and Human Interleukin-10 (IL-10) ELISA kits (E-EL-H6154). Yeasen Biotech Co., Ltd. supplied the PAGE gel fast preparation kit (Catalog number: 20325ES), while Abbkine supplied the ultra-sensitive ECL luminous liquid (Catalog number: BMU102-CN). We purchased the SYBR® Green Premix Pro Taq HS qPCR Kit II (Catalog number: AG11702) and the Evo M-MLV Mix Kit with gDNA Clean for qPCR reagents (Catalog number: AG11728) from Hunan Accurate Biotechnology. Sigma supplied the lipopolysaccharide (LPS) (L2880, St. Louis, USA), and MedChemExpress the agar (AG490, HY-12,000, Monmouth Junction, NJ, USA). From the Jiangsu Provincial Traditional Chinese Medicine Hospital, we acquired the traditional Chinese medicines Xianhecao and Huanglian. These were soaked, decocted, filtered, and concentrated to 1 g/mL in our hospital’s preparation room for use in future research, and then put in a freezer set to -80 °C.

### Experimental Animals

A total of seventy SPF male C57BL/6 N mice, aged 9–12 weeks, with a body weight of (22 ± 2) g, originated from the Vital River Laboratory Animal Technology Co., Ltd. in Beijing, with the following license number: SCXK (Beijing) 2021-0006. The mice were given free reign over their nourishment plus water sources.

### Modeling, Grouping, and Drug Administration

**Fig. 1:**
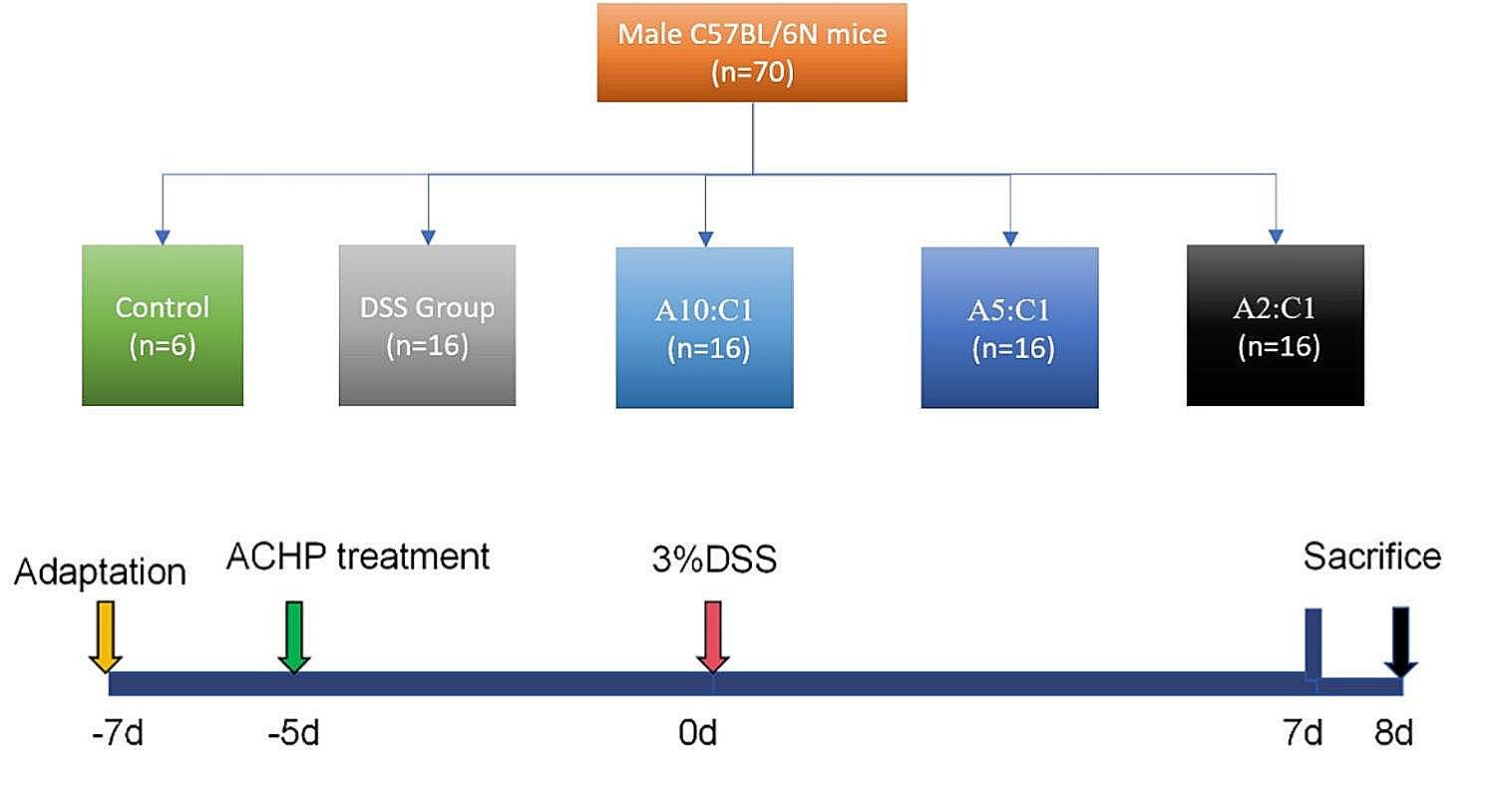
Schematic diagram of experimental design. After one week of adaptation, the mice were randomly divided into five groups: Control group, DSS group, A10: C1 group, A5: C1 group, and A2: C1 group. DSS group: Drinking water containing 3% DSS. Traditional Chinese medicine was administered orally to each proportion group 5 days before modeling, with Xianhecao-Huanglian Drug Pair decoction until the end of the experiment. Water containing 3% DSS was freely obtained for one week. Among them, A10: C1 group Xianhecao: Huanglian = 10:1 (w/w), A5: C1 group Xianhecao: Huanglian = 5:1 (w/w), A2: C1 group Xianhecao: Huanglian = 2:1 (w/w)

The mice were then divided into five separate groups: one set of controls (*n* = 6), a DSS group (*n* = 16), a group receiving a 3% DSS + Xianhecao-Huanglian drug pair in a 10:1 ratio (referred to as A10:C1; with Xianhecao administered at 5.45 g/kg/d and Huanglian at 0.55 g/kg/d, *n* = 16), a group receiving a 3% DSS + Xianhecao-Huanglian drug pair in a 5:1 ratio (referred to as A5:C1; with Xianhecao administered at 5 g/kg/d and Huanglian at 1 g/kg/d, *n* = 16), and a group receiving a 3% DSS + Xianhecao-Huanglian drug pair in a 2:1 ratio (referred to as A2:C1; with Xianhecao administered at 4 g/kg/d and Huanglian at 2 g/kg/d, *n* = 16). After a week of adaptive feeding, control, and DSS group mice had free access to water and a normal diet. Treatment groups received preventive intervention for 5 days, starting on the third day of adaptive feeding. From day 7, DSS and treatment groups had free access to a 3% DSS solution, while the treatment group continued traditional Chinese medicine intervention for 7 days. Mice were euthanized 8 days post-decapitation, as illustrated in Fig. 1. A daily check was made on the mice’s weight throughout this timeframe. Following the experiment, in C57BL/6 N mice Whole blood was acquired through orbital blood collection using a heparin sodium anticoagulant tube. After centrifugation at 4 ℃, 3000 rpm/min for 10 min, the obtained serum was stored at -80 ℃ for testing. Mice were euthanized by decapitation, and prompt dissection provided colon samples. The colon length was measured, and after washing feces, it was opened longitudinally, rinsed with pre-cooled saline, and measured without external force. LCM of the obvious lesion was taken, frozen in liquid nitrogen, and then transferred to a -80 ℃ refrigerator for storage, Retain the obvious lesion LCM and fix it in 4% paraformaldehyde for 24 h, followed by making pathological sections for the purpose of conducting staining with hematoxylin and eosin (HE), periodic acid-Schiff (PAS) analysis, immunohistochemistry, and immunofluorescence. Extract the spleen of the mouse, dry the residual blood with filter paper, and weigh. In the case of rats, fasted for 12 h before the last administration and couldn’t help but water. Following the last gavage, mice were anesthetized with pentobarbital sodium (SIGMA company, USA) for 1 h. Blood was collected from the abdominal aorta and allowed to stand for 1 h. Centrifugation at 3000 rpm for 10 min yielded upper clear liquid, combined with the same serum group. This mixture was subjected to complement inactivation at 56 ℃ in a water bath for 30 min. Subsequently, the serum underwent filtration and sterilization using a 0.22 µ m microporous filter membrane within a sterile ultra-clean workbench. After packaging, it was sealed and stored in a -80 ℃ refrigerator for future use.

### Drug-Contained Serum Formulation

A total of twenty SPF male SD rats weighing 200 ± 20 g, were segregated to split into two categories: the serum groups that did not include any drugs and those that did both, each consisting of ten rats. These rats were obtained under license number SCXK (Beijing) 2021–0011 via the Beijing-based Vital River Laboratory Animal Technology Co., Ltd. The optimal drug pair ratio of 5:1 was selected based on prior experimental outcomes to prepare the decoction of traditional Chinese medicine. The medicated group was orally administered with a Xianhecao-Huanglian drug pair medicinal decoction at a dose of 3.24 g/kg per day, in contrast to the blank serum group, which was given the same amount of saline twice da, for four consecutive days. One hour following the final administration, blood samples were collected from the rats’ abdominal aorta, centrifuged, complement-inactivated, sterilized via filtration, and stored for subsequent cellular experiments.

### Colon Histological Analysis

Colonic tissue samples were extracted, rinsed with PBS, and preserved in a 4% paraformaldehyde solution for at least 24 h. After dehydration in an ethanol gradient, the slices of 5 μm were cut from tissues that had been fixed in paraffin. The samples were stained using the standard method of H&E., and after sealing, the structural integrity of the colon was examined under a microscope. Periodic Acid-Schiff (PAS) staining, following established protocols, was carried out to evaluate goblet cell growth and mucus secretion.

### Disease Activity Index (DAI) and Histological Evaluation

During the experiment, body weight, stool character, and fecal occult blood were recorded. The disease activity index (DAI) was calculated based on the scoring system [17] and histological evaluation [18] in accordance with previously published methods (Table 1).

**Table 1:** The Disease Activity Index (DAI) score

Score	Weight loss (%)	Stool character	Fecal occult blood
0	0	Normal formed	Negative
1	1–5%		
2	5–10%	Loose stool	Positive
3	10–20%		
4	>20%	Diarrhea	Gross bleeding

### Immunohistochemistry (IHC) Staining of Colonic Tissue

Sections of paraffin-embedded tissue were processed for routine deparaffinization, followed by antigen retrieval, endogenous peroxidase inhibition, and sealing. Subsequently, the pieces were incubated with primary antibodies, Muc2 (1: 2000), Occludin (1:250), ZO-1 (1:1000), and Claudin-1 (1:250), at 4 °C for the night., followed by secondary antibodies and DAB staining. The nuclei of the cells were counterstained with hematoxylin. The slices were dehydrated, mounted using neutral glue, dried out, and then taken and viewed under a microscope.

### Imaging Using Fluorescence Microscopy

Immunofluorescence staining was conducted on paraffin-embedded sections of colonic tissue. After deparaffinization, sections of paraffin were soaked and then the extraction of the antigen was performed in a citrate buffer (pH 6.0). To minimize endogenous peroxidase operation, 3% H2O2 was added. After applying serum blocking, the samples were incubated overnight at 4 °C using primary antibodies that were properly diluted. The cell nuclei were stained with DAPI, and then fluorescent secondary antibodies were added. After 5 min of incubation under light-protected conditions, the slices were carefully sealed, examined, and photographed under a microscope.

### Cell Culture and Treatment

NCM460 intestinal epithelial cells and LOVO colon cancer cells were procured from Wuhan Procell and cultured in RPMI-1640 and Ham’s F-12 K media, respectively, both incubated at 37 °C with 5% CO2 and contained 10% FBS as well as 1% penicillin/streptomycin. The cells were depleted of serum for 24 h before every test. By incubating LoVo as well as NCM460 cells with 10 µg/mL of LPS, an inflammatory cell model for IBD was created.Cells were treated with varying concentrations of Xianhecao-Huanglian drug pair medicated serum (0%, 10%, 20%, 30%, 40%, and 50%) for 24 h to investigate the effect of the drug-loaded serum on cell survival rates. First, the cells were treated with 20% medicated serum and compound AG490, either individually or in combination. The medicated serum was added 2 h prior to AG490, followed by 24 h of LPS stimulation at 37 °C. The influence of Xianhecao-Huanglian drug pair medicated serum on LOVO and NCM460 cells under LPS stimulation was assessed.

### Monitoring the Survival of Cells

The amount of cells planted in a 96-well plate was 1 × 105 cells/mL, with 100 µL of medium per well. They were allowed to adhere overnight. Different concentrations of Xianhecao-Huanglian drug pair medicated serum were introduced into their corresponding wells, after which LPS was added to the cells, either individually or in combination, for 24 h. When the procedure was finished, 10 µL of CCK-8 solution was transferred to every well and allowed to incubate for another 2 h in the incubator. A plate reader was used to detect absorbance around 450 nm in order to ascertain the vitality of the cells.

### ELISA

Mouse colonic tissue was collected, weighed, homogenized in PBS, and then centrifuged to obtain the supernatant. A 6-well plate was used to seed the cells, which were cultivated overnight at a density of 1 × 10^5^ cells/mL in 1 mL of media per well. The ELISA kit’s instructions were followed to quantify the amounts of TNF-α, IL-10, IL-1β, as well as IL-6 in the obtained tissues, blood, and cell supernatants from mice.

### RT-qPCR

After deciding on the RNA content, the entirety of RNA was isolated through colonic tissues. Then, utilizing the Evo M-MLV RT Mix Kit with gDNA Clean for qPCR (AG11728, ACCURATEBIOTECHNOLOGY, HUNAN, Co., Ltd.), cDNA was produced employing reverse transcription. Afterward, the cDNA was produced for RT-qPCR amplification by the SYBR® Green Premix Pro Taq HS qPCR Kit II (AG11702, ACCURATE BIOTECHNOLOGY, HUNAN, Co., Ltd.). The 2-ΔΔCt technique was used for data analysis, with β-actin acting as the internal reference. In Table 2 you can find the primer sequences that were needed for this study.

**Table 2:** The primers information of genes in this study

Gene name	Sequence
IL-Iβ-F	5´-TGCCACCTTTTGACAGTGATG-3´
IL-Iβ-R	5’-AAGGTCCACGGGAAAGACAC-3’
TNF-α-F	5’-AGGCACTCCCCCAAAAGATG-3’
TNF-α-R	5’-TGGTGGTTTGTGAGTGTGAGG-3’
IL-6-F	5’-CAACGATGATGCACTTGCAGA-3’
IL-6-R	5’-GTGACTCCAGCTTATCTCTTGGT-3’
IL-10-F	5’-GCTCCAAGACCAAGGTGTCT-3’
IL-10-R	5’-CGGAGAGAGGTACAAACGAGG-3’
β-actin primer-F	5’-CTAGGCGGACTGTTACTGAGC-3’
β-actin primer-R	5’-ATGTTTGCTCCAACCAACTGC-3’

### Western Blot

Protein extraction was performed on cells as well as the mouse colonic tissue using a lysis buffer containing RIPA (Beyotime, China), along with proteinase as well as phosphatase inhibiting agents. The amount of protein was determined using the BCA kit. 20 µg of each protein was poured into each well, and SDS-PAGE gel electrophoresis was performed on eighty volts to distinguish the intended proteins. The concentration of the gel used for SDS-PAGE was 8%, 12.5%, and 15%. Afterward, protein fragments were moved to a PVDF membrane from Millipore. The cell membrane was blocked using 5% skim milk at ambient temperature over 1 h, then subjected to an overnight soak at 4 °C with particular primary antibodies. The antibodies used in this study comprised ATG12, p62, LC3A/B, Beclin-1, STAT3, p-STAT3, ZO-1, MUC2, β-actin, and β-Tubulin, each at a 1:1000 dilution, while JAK2 and p-JAK2 were utilized at a 1:500 dilution ratio. On the next day, the membrane was carefully washed multiple times via PBST. Then, HRP-conjugated goat anti-rabbit or anti-mouse IgG (1:5000, 111-035-003/115-035-003; Jackson) was put on and incubated at ambient temperature for 1 h. After rinsing with TBST, we proceeded with ECL development as well as exposure. The grayscale measurements for every single protein band were subsequently evaluated using Image J software, just like a biologist would do.

### Data Analysis

For every circumstance, a minimum number of three separate studies were conducted, as well as the outcomes are displayed as the mean ± standard deviation. The statistical significance was evaluated using an analysis of variance (ANOVA) for comparing each group, and then Tukey’s t-test was used for post hoc evaluation. The statistical evaluation was performed employing GraphPad Prism version 9.5.0 (GraphPad Software Inc 9.5.0). Statistical significance was determined by a p-value lower than 0.05.

## Results

### Effect of Xianhecao-Huanglian Drug Pair on Improving DSS-Induced IBD Symptoms

To validate the role of the Xianhecao-Huanglian drug pair in IBD, an acute IBD mouse model was established using 3% DSS induction (Fig. 1).

**Fig. 2:**
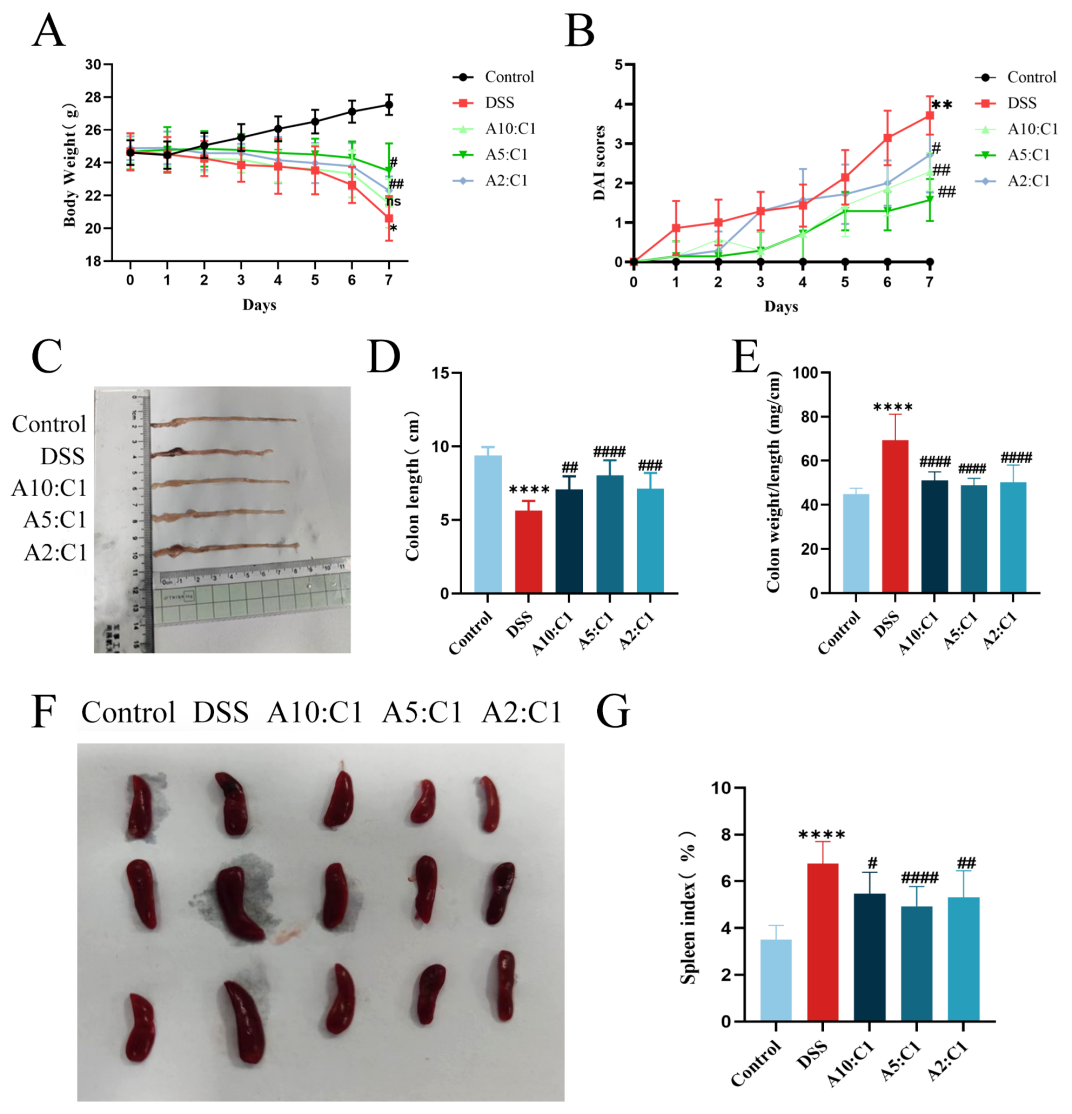
Xianhecao-Huanglian Drug Pair alleviates DSS-induced colitis. **A** Body weight changes. **B** DAI scores. **C** and **D**. Colon length. **E**. The ratio of colon weight to length (mg/cm). **F**. Spleen. **G**. Spleen index. The calculation method for the spleen coefficient is to divide the spleen mass by the body mass of the mouse. Data are expressed as the mean ± SD, (*n* = 5–10). Compared with control group, * *p* < 0.05, * * *p* < 0.01, * * ** *p* < 0.0001; Compared with DSS group, # *p* < 0.05, # # *p* < 0.01, # ## *p* < 0.001, # # ##*p* < 0.0001; ns, not significant

The DSS-treated mice displayed significant clinical signs of acute colitis, such as loose stools, rectal bleeding, noticeable weight loss (Fig. 2A), and a gradual increase in DAI score (Fig. 2B) over time, confirming the successful establishment of the model. As depicted in Fig. 2C-E, DSS-induced colon shortening was markedly alleviated after treatment with the Xianhecao-Huanglian drug pair, with a more pronounced effect observed when they were combined in a 5:1 ratio. The spleen, being a crucial immune organ, often enlarges during inflammation. We observed that Xianhecao-Huanglian drug pair treatment may influence mouse immunity. Following euthanasia, spleen weights were measured, and the organ index was calculated. Notably, the spleen index of the DSS-treated mice experienced a notable increase in comparison to the control group, while it decreased significantly after treatment with the drug pair, demonstrating statistical significance (Fig. 2F-G). In summary, these findings indicate that the Xianhecao-Huanglian drug pair has a protective effect against DSS-induced IBD.

**Fig. 3:**
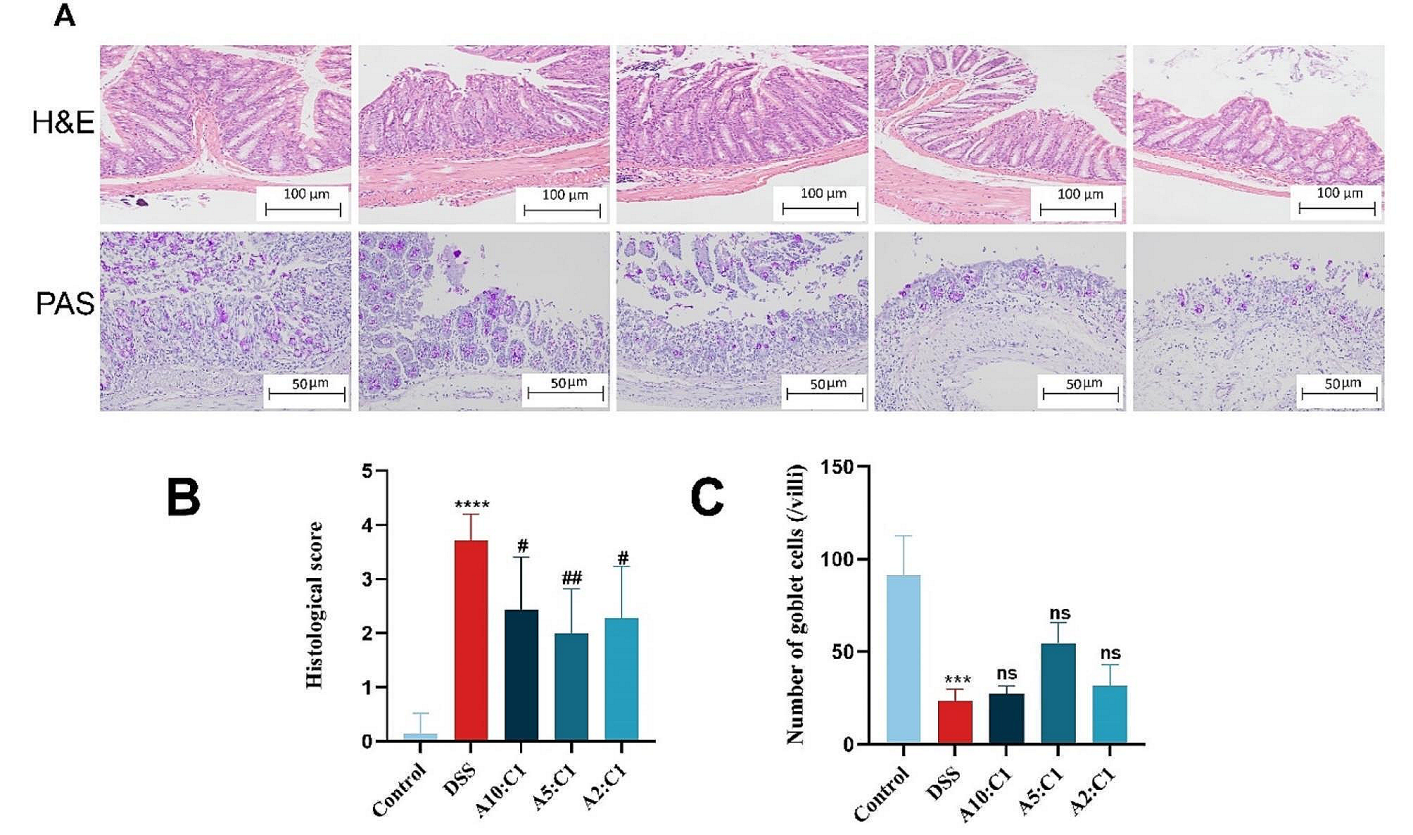
The effect of the Xianhecao-Huanglian Drug Pair on the pathological changes of colon tissue. **A**. Colon tissue staining using H&E (enlarged, 200× scale bar = 100 μm) and representative staining photos of PAS (enlarged, 200× scale bar = 50 μm). **B** Histological score. **C**. Number of goblet cells. Data are expressed as the mean ± SD, (*n* = 3–6). Compared with control group, * * * *p* < 0.001, and * * * *p* < 0.0001; Compared with DSS group, # *p* < 0.05, # # *p* < 0.01, ns, not significant

The histopathological examination additionally validated the pathological alterations induced by DSS. As depicted in Fig. 3A, HE staining illustrated that the control group exhibited nearly normal colonic tissue characterized by an intact colonic tissue structure, well-preserved crypt structures, orderly gland arrangement, and observable goblet cells, devoid of indications of inflammatory cell infiltration. In contrast, the model group displayed severe disruption in colonic wall structure, significant damage to goblet cells and crypts, disrupted glandular structures, surface epithelial erosion, and substantial inflammatory cell infiltration within colonic tissue, resulting in a higher histological score (Fig. 3B). Post-treatment with the drug pair gradually restored the pathological damage of colonic tissue and reduced the inflammatory cell infiltration, significantly ameliorating the morphopathological changes induced by DSS and lowering the histological score.

### Effects of the Xianhecao-Huanglian Medicine Combination on Mucin Along with Tight Junction Protein Expression

The molecules forming tight junctions (TJs) among epithelial cells contribute significantly to maintaining a healthy gut mucosal barrier, closely associated with the progression and development of IBD. The colonic tissue’s mucin MUC2, secreted by goblet cells, is pivotal in forming a mucus layer on the intestinal surface, which shields the intestine from noxious substances and maintains intestinal homeostasis [19, 20].

**Fig. 4:**
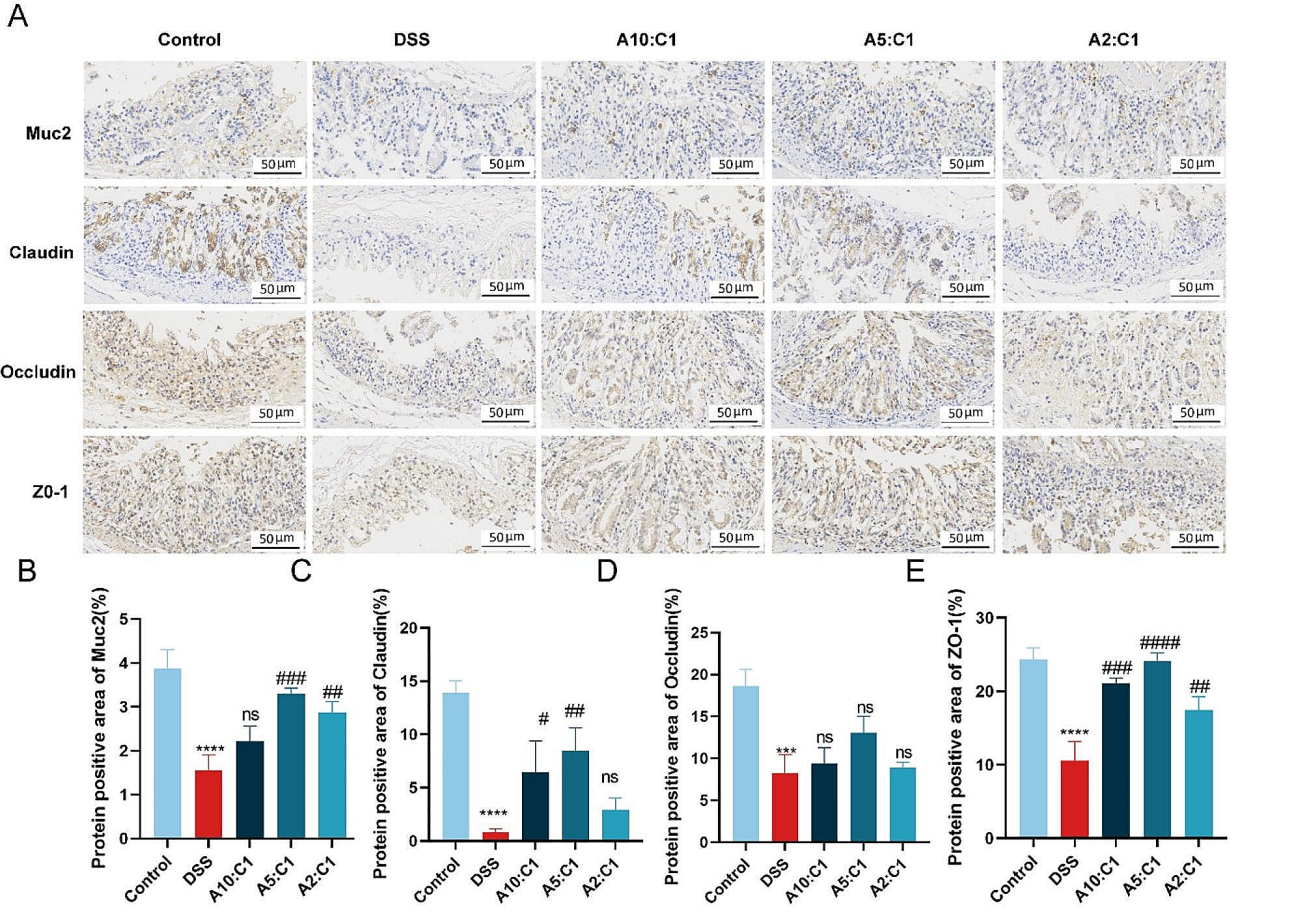
Xianhecao-Huanglian Drug Pair protects intestinal barrier function. **A**. Representative immunohistochemical staining images of MUC2, Occludin, ZO-1, and Claudin-1 in colon tissues of different groups of mice (enlarged, 400×, Scale bar = 50 μm). **B-E**. Positive area ratio of MUC2, Claudin-1, Occludin, and ZO-1 in colon tissue of different groups of mice. Data are expressed as the mean ± SD, (*n* = 3). Compared with control group, * * * *p* < 0.001, and * * * * *p* < 0.0001; Compared with DSS group, # *p* < 0.05, # # *p* < 0.01, # # # *p* < 0.001, # # # # *p* < 0.0001; ns, not significant

Consequently, we measured the levels of TJ proteins, such as Claudin-1, Occludin, ZO-1, and mucin MUC2, through immunohistochemistry. As displayed in Fig. 4A-E, mice administered DSS had lower levels of TJ protein expression in colonic regions as contrasted with controls. There was a significant decrease in goblet cell numbers, severe mucus layer damage, and a substantial reduction in mucin MUC2. Conversely, in the drug pair groups, the levels of Claudin-1, Occludin, while ZO-1 were more similar to those in the group receiving a healthy control. The decrease in goblet cell numbers (Fig. 3A and C) and the loss of mucin MUC2 were significantly ameliorated.

### Inhibition of DSS-induced Secretion of Pro-inflammatory Cytokines by Xianhecao-Huanglian Drug Pair

**Fig. 5:**
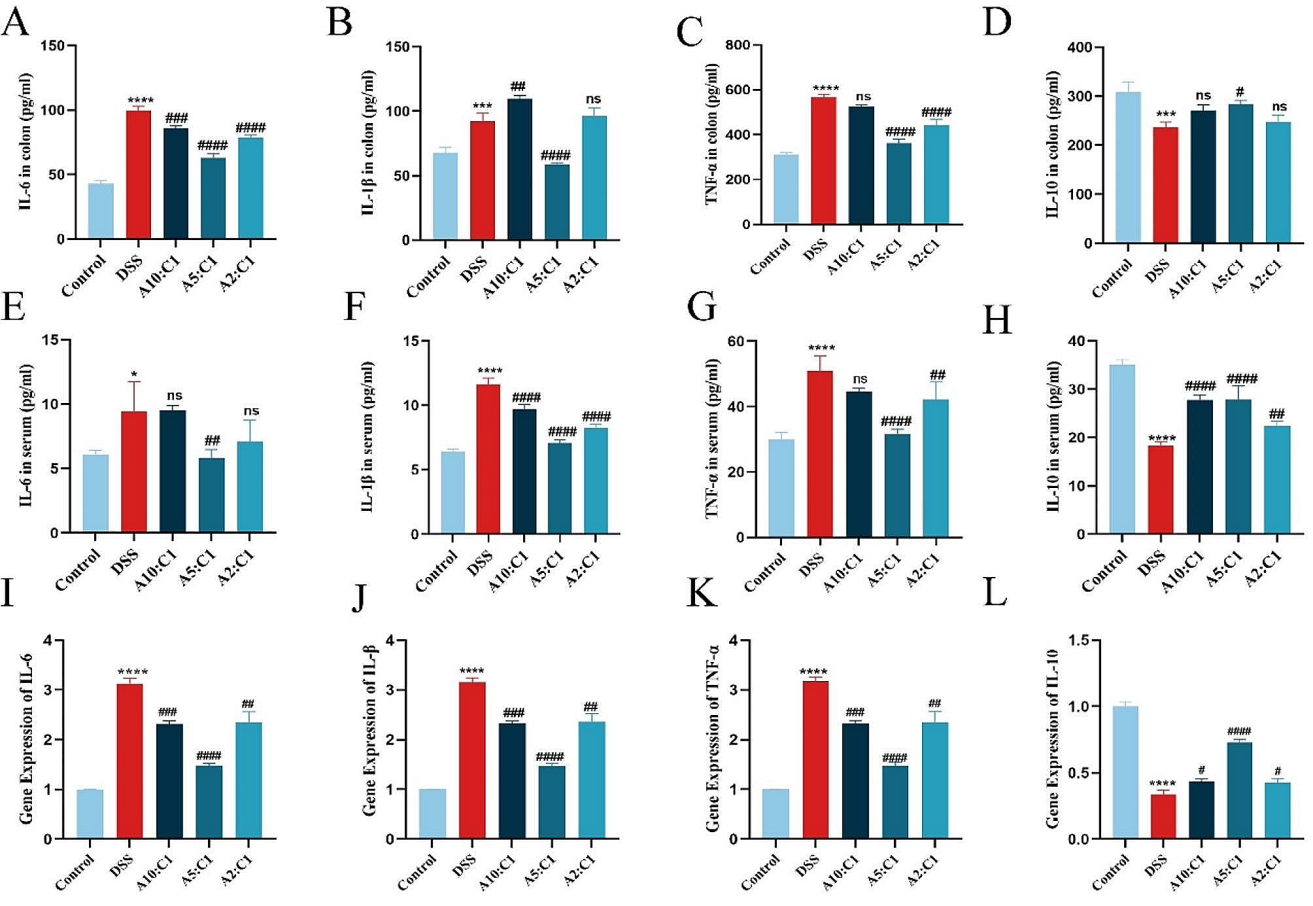
The combination of the Xianhecao-Huanglian Drug Pair can alleviate the inflammatory response caused by DSS. **A-L**. Elisa reagent kit for detecting IL-6 and IL-1 in colon tissue β、TNF- α and the content of IL-10. **E-H**. Elisa reagent kit for detecting IL-6 and IL-1 in mouse serum β、TNF- α And the content of IL-10. **I-L**. RT-QPCR determination of IL-6 and IL-1 in mouse colon tissue β、TNF-α and the mRNA level of IL-10. Control: Normal drinking water. DSS group: Drinking water containing 3% DSS.A10: C1: Xianhecao-Huanglian Drug Pair 10:1 group, Xianhecao 5.45 g/kg, Huanglian 0.55 g/kg; A5: C1: Xianhecao-Huanglian Drug Pair 5:1 group, Xianhecao 5 g/kg, Huanglian 1 g/kg; A2: C1: Xianhecao-Huanglian Drug Pair 2:1 group; Xianhecao 4 g/kg, Huanglian 2 g/kg. Data are expressed as the mean ± SD, (*n* = 3). Compared with control group, * *p* < 0.05, * * * *p* < 0.001, and * * * *p < 0.0001; Compared with DSS group, # *p* < 0.05, # # *p* < 0.01, # # # *p* < 0.001, # # # # *p* < 0.0001; ns, not significant

Inflammation is a prominent characteristic of IBD, where A rise in the expression of cytokines that promote inflammation and a reduction in cytokines that inhibit inflammation play crucial roles in its pathogenesis [21, 22]. IL-6, TNF-α, IL-1β, and IL-10 serve as pivotal pathological mediators in inflammatory bowel disease. Thus, we used ELISA to detect the levels of cytokines that are pro-inflammatory TNF-α, IL-1β, as well as IL-6, as well as the cytokine that fights inflammation, IL-10, in both colonic tissues and blood. Figure 5A-D shows that in contrast with the control group, the model mice showed a notable rise in the production of inflammatory cytokines such as IL-1, IL-6, and TNF-a in colonic tissues, and a reduction in the amount of the cytokine that prevents inflammation, IL-10. The levels of TNF-α, IL-1β, and IL-6 in the gastrointestinal tissues fell dramatically after therapy with the medication pair, whereas the level of IL-10 rose. This trend was also observed in the mouse serum (Fig. 5E-H). These results were further validated using qQPCR (Fig. 5I-L). These findings suggest that the Xianhecao-Huanglian drug pair can mitigate the inflammatory response induced by DSS.

### Xianhecao-Huanglian Drug Pair Promotes Inhibition of Autophagy in DSS-induced IBD Mice via the JAK2/STAT3 Signaling Pathway

**Fig. 6:**
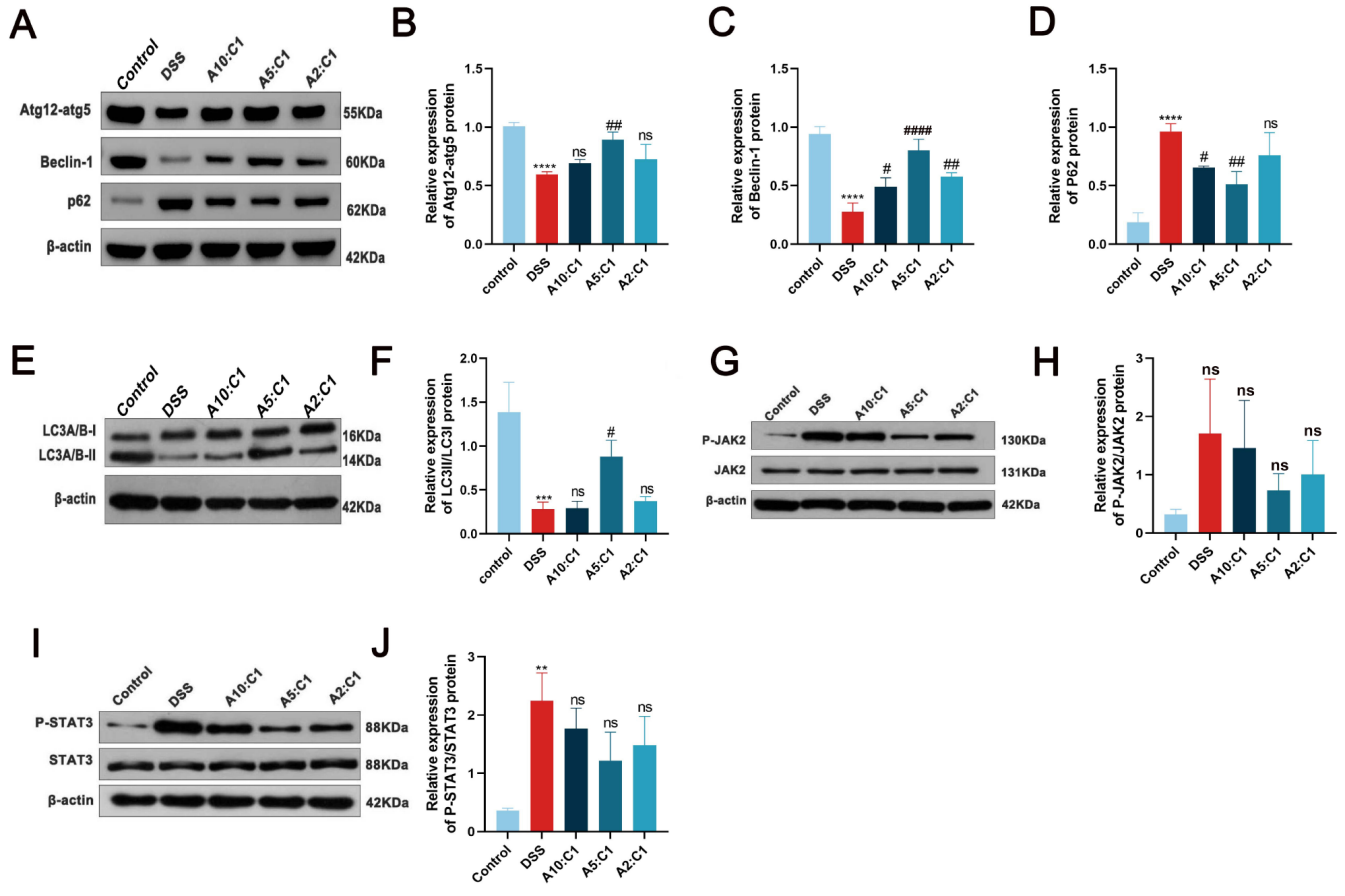
Xianhecao-Huanglian Drug Pair promotes the inhibition of autophagy in DSS-induced IBD mice and activates the JAK 2/STAT 3 signaling pathway. **A**. Western blot was used to detect the expression of Atg12-atg5, Beclin-1, and p62 proteins. **B-D**. The relative expression levels of Atg12 atg5, Beclin-1, and p62 proteins. **E**. Western blot was used to detect the expression of LC3II/I proteins. **F**. The relative expression levels of LC3II/Iproteins. **G**.Western blot was used to detect the phosphorylation levels of JAK2 . **H**.The relative expression levels of phosphorylated JAK2 was normalized to JAK2. **I**.Western blot was used to detect the phosphorylation levels of STAT3. **J**. The relative expression levels of phosphorylated STAT3 was normalized to STAT3.Control: Normal drinking water. DSS group: Drinking water containing 3% DSS. A10: C1: Xianhecao-Huanglian Drug Pair 10:1 group, Xianhecao 5.45 g/kg, Huanglian 0.55 g/kg; A5: C1: Xianhecao-Huanglian Drug Pair 5:1 group, Xianhecao 5 g/kg, Huanglian 1 g/kg; A2: C1: Xianhecao-Huanglian Drug Pair 2:1 group; Xianhecao 4 g/kg, Huanglian 2 g/kg. Data are expressed as the mean ± SD, (*n* = 3). Compared with control group, * *p* < 0.05, * * *p* < 0.01, * * * *p* < 0.001, and * * * *p* < 0.0001; Compared with DSS group, # *p* < 0.05, # # *p* < 0.01, # # *p* < 0.001, # # *p* < 0.0001; ns, not significant

To further explore the impact of the Xianhecao-Huanglian drug combination on autophagy in DSS-induced IBD mice, we examined the expression of proteins associated with autophagy, including ATG12, Beclin-1, LC3A/B, and p62 (Fig. 6A-F), in colitis tissues using Western blot analysis. When comparing the control group to the model group, there was an apparent reduction in the protein levels of LC3A/B, Beclin-1, as well as ATG12. On the other hand, the expression of p62 protein increased. Compared to the control group, the one that received the treatment with the Xianhecao-Huanglian drug pair resulted in varying degrees of upregulation in ATG12, Beclin-1, and LC3A/B levels, and a reduction in p62 protein expression, with the A5:C1 group demonstrating the most pronounced changes.

**Fig. 7:**
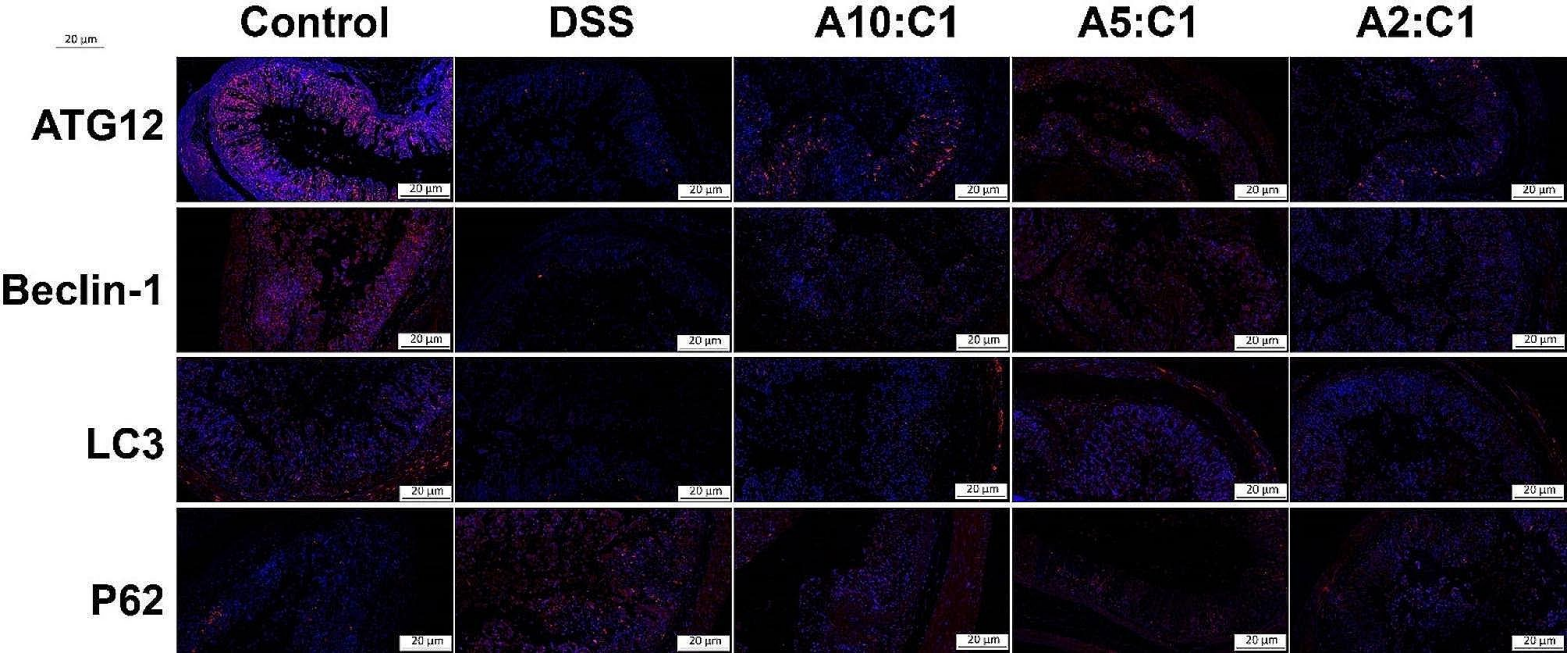
Representative immunofluorescence staining images of colon tissues stained with ATG12, Beclin-1, LC3A/B, and P62 in different groups of mice (magnification, 200×, Scale bar = 20 μm). The nucleus was stained with DAPI (blue). Control: Normal drinking water. DSS group: Drinking water containing 3% DSS. A10: C1: Xianhecao-Huanglian Drug Pair 10:1 group, Xianhecao 5.45 g/kg, Huanglian 0.55 g/kg; A5: C1: Xianhecao-Huanglian Drug Pair 5:1 group, Xianhecao 5 g/kg, Huanglian 1 g/kg; A2: C1: Xianhecao-Huanglian Drug Pair 2:1 group; Xianhecao 4 g/kg, Huanglian 2 g/kg. Data are expressed as the mean ± SD, (*n* = 3)

Further confirmation through immunofluorescence staining revealed that, as seen in Fig. 7, in contrast to the group that served as a model, the fluorescence intensity of ATG12, Beclin-1, and LC3A/B expression increased to varying degrees, indicating that the Xianhecao-Huanglian drug pair enhances autophagy that is inhibited by DSS-induced IBD. The route for signaling by JAK2/STAT3, a widely studied pathway in various inflammatory diseases, is activated in IBD mice. Examination of JAK2, STAT3, p-JAK2, as well as p-STAT3 expression of proteins using Western blotting, as shown in Fig. 6G-J, demonstrated that the Xianhecao-Huanglian drug pair in varying degrees reduced JAK2 as well as STAT3 phosphorylation levels, which had been increased by DSS. These results indicate that the drug pair promotes autophagy in DSS-induced IBD mice by means of the JAK2/STAT3 signaling route.

### Impact of Xianhecao-Huanglian Drug Combination on LPS-Stimulated Cell Viability in LOVO and NCM460

**Fig. 8:**
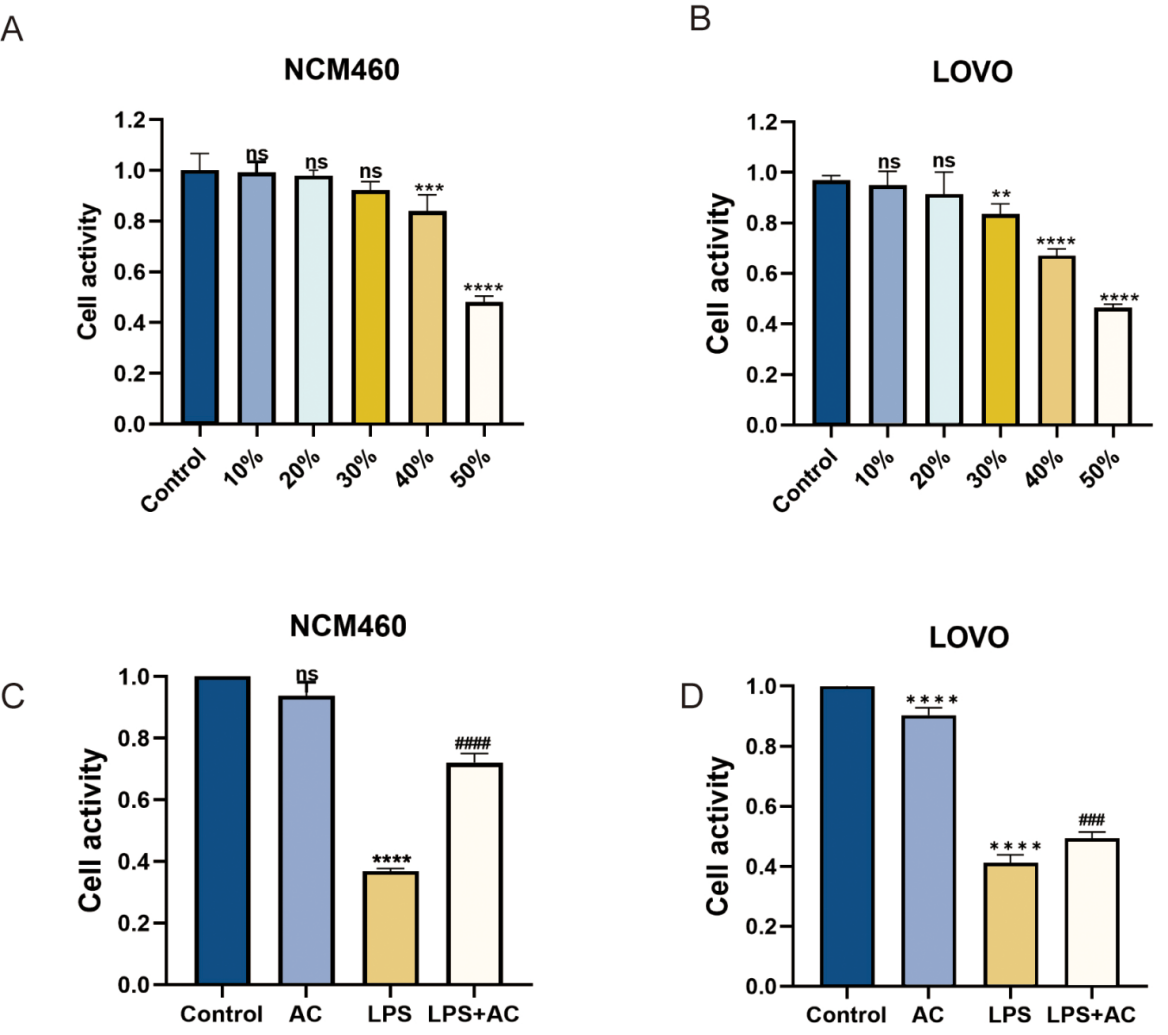
The effect of ACHP-DS on the viability of NCM460 and LOVO cells under LPS stimulation. **A** and **B**. The CCK-8 method was used to detect the effects of different concentrations (0, 10, 20, 30, 40, and 50%) of ACHP-DS on the viability of NCM460 and LOVO cells. **C** and **D**. After LPS and ACHP-DS treatment of cells, the CCK-8 method was used to evaluate cell viability. Control: Blank serum treated cells; LPS: LPS (10ug/ml) treated cells; AC: 20% ACHP-DS treated cells; LPS + AC: 20% ACHP-DS pretreated cells for 2 h and co-incubated with LPS (10ug/ml) for 24 h. Data are expressed as the mean ± SD, (*n* = 3). Compared with control group, * * *p* < 0.01, * * * *p* < 0.001, and * * * *p < 0.0001; Compared with LPS group, # *p* < 0.05, # # *p* < 0.01, # # # *p* < 0.001, # # # # *p* < 0.0001; ns, not significant

To investigate the impact of the Xianhecao-Huanglian drug pair on the potential for LOVO with NCM460 cell survival, cells were exposed to Xianhecao-Huanglian drug pair-containing serum at varying concentrations (0%, 10%, 20%, 30%,40% and 50%). The control group was represented by the blank serum group, and The CCK-8 assay was used to determine the cell viability. As displayed in Figs. 8A-B and 20% serum containing the Xianhecao-Huanglian medication combination did not affect the two cell lines’ survival, and there was no statistically significant difference between the experimental group as well as the control arm. Nevertheless, as the serum concentration increased, cell viability gradually decreased. Therefore, the optimal treatment concentration of Xianhecao-Huanglian drug pair-containing serum for subsequent experiments was determined to be 20%. Further investigation was carried out to investigate the effects of the Xianhecao-Huanglian drug pair on the viability of LOVO and NCM460 cells under LPS stimulation. As shown in the Fig. 8C-D 10 µg/mL of LPS significantly inhibited cell proliferation. In comparison to the LPS group, the Xianhecao-Huanglian drug pair-containing serum significantly increased cell viability, with a notable difference observed. It was also noted that using Xianhecao-Huanglian drug pair serum alone had no impact on cell viability.

**Fig. 9:**
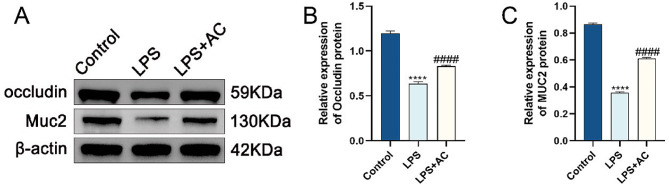
ACHP-DS can improve intestinal barrier dysfunction in LPS-induced IBD cell model. **A** Western blotting images of occludin, Muc2 protein expression. **B** The relative expression level of Occludin protein **C** The relative expression level of Muc2 protein. Control: Blank serum treated cells; LPS: LPS (10ug/ml) treated cells; LPS + AC: 20% ACHP-DS pretreated cells for 2 h and co-incubated with LPS for 24 h. Data are expressed as the mean ± SD, (*n* = 3). Compared with control group, * * * * *p* < 0.0001; Compared with LPS group, # # # #*p* < 0.0001; ns, not significant

This suggests that the Xianhecao-Huanglian drug pair may provide protection against DSS-induced IBD by preserving the integrity of the epithelial and mucosal barriers. In vitro, experimental data (Fig. 9A-C) confirmed the substantial upregulation of the suppressed expression of Occludin and Muc2 proteins in the inflammatory model cells after exposure to Xianhecao-Huanglian drug pair-containing serum. In conclusion, the Xianhecao-Huanglian drug pair may boost the production of MUC2 as well as Occludin, two proteins that form tight junctions in the intestine, thereby alleviating intestinal barrier dysfunction.

### Xianhecao-Huanglian Drug Pair Modulates the JAK2/STAT3 Signaling Pathway to Inhibit LPS-Stimulated Cell Inflammation

**Fig. 10:**
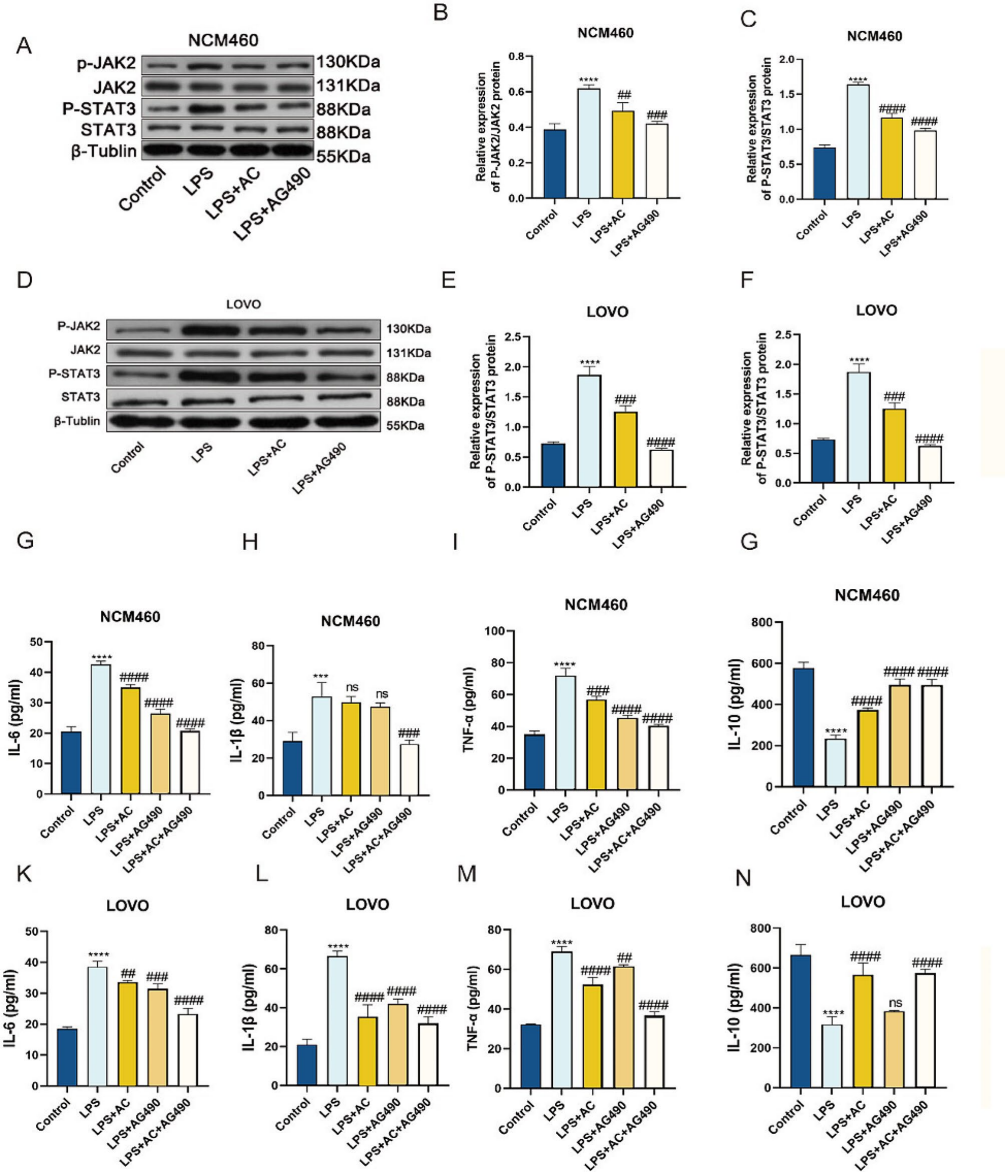
ACHP-DS reduces LPS-induced cellular inflammatory response through the JAK2/STAT3 signaling pathway. **A**. Western blot was used to detect the phosphorylation levels of JAK2 and STAT3 in NCM460 cells. **B-C**. The relative expression levels of phosphorylated JAK2 and STAT3 were normalized to JAK2 and STAT3, respectively. **D**. Western blot was used to detect the phosphorylation levels of JAK2 and STAT3 in LOVO cells. **E**-**F**. The relative expression levels of phosphorylated JAK2 and STAT3 were normalized to JAK2 and STAT3, respectively. **G**-**N**.ELISA method for detecting TNF- α、IL-1 β、IL-6 and IL-10 content. Control: Cells treated with 20% blank serum; LPS: 10ug/ml of LPS; LPS + AC: 20% ACHP-DS pretreated cells for 2 h, then co-incubated with 10ug/ml LPS for 24 h; LPS + : LPS Before stimulation, use AG 490 (10 µM) Pre-treat NCM460 and LOVO cells for 1 h; LPS + AG490 + AC: Using AG 490 Pre-treat NCM460 and LOVO cells for 1 h, pre-treat NCM460 and LOVO cells with 20% ACHP-DS for 2 h, and add LPS Incubate together for 24 h. Data are expressed as the mean ± SD, (*n* = 3). Compared with control group, * * * *p* < 0.001, and * * * * *p* < 0.0001; Compared with LPS group, # # *p* < 0.01, # # #*p* < 0.001, # # *p* < 0.0001; ns, not significant

To investigate whether the Xianhecao-Huanglian drug pair-mediated inflammation is linked to the route for signaling via JAK2/STAT3, the phosphorylation of JAK2 and STAT3 proteins in the LPS-induced IBD inflammatory cell model was examined using the Western blot technique. Xianhecao-Huanglian along with AG490 were able to reduce the elevated phosphorylation of JAK2 as well as STAT3 which LPS caused, as shown in Fig. 10A-F. One possible explanation for the anti-inflammatory effects of the Xianhecao-Huanglian medication combination is that it inhibits the phosphorylation of JAK2 and STAT3 by modulating the JAK2/STAT3 signaling pathway. In the supernatant, the levels of pro-inflammatory cytokines IL-6, IL-1β, as well as TNF-α rose considerably after LPS stimulation, according to ELISA data, although IL-10 levels dropped. Pretreatment with the AG490 inhibitor and the Xianhecao-Huanglian medication combo significantly reduced LPS-induced levels of pro-inflammatory cytokines and increased IL-10 expression, two cytokines that fight inflammation. It is noteworthy that the combined use of both drugs exhibited a more potent anti-inflammatory effect than each drug alone. These findings suggest that the Xianhecao-Huanglian drug pair suppresses cell inflammation stimulated by LPS via the signaling pathway that involves JAK2 and STAT3.

### Combination Serum Including Xianhecao as Well as Huanglian drugs Attenuated Cell Pro-inflammatory Cytokine Production in Response to DSS

The serum containing the Xianhecao-Huanglian drug pair reduced the generation of cytokines that promote inflammation induced by DSS in cells. In order to investigate how the serum comprising the Xianhecao-Huanglian medication combination affected the inflammatory reaction in cells challenged with LPS, the ELISA was used to identify the levels of IL-10, TNF-α, IL-1β, and IL-6 in the cultured cell supernatant. With the use of blank serum, a group serving as a control was formed. The experimental group showed a significant rise in TNF-α, IL-1β, and IL-6 expression after stimulation with LPS, as shown in Fig. 10G-N, plus a significant drop in IL-10 expression. Be that as it may, the amount of IL-10 was up whereas TNF-α, IL-1β, and IL-6 were decreased after treatment using the serum that included the Xianhecao-Huanglian medication combination. This aligns with the anti-inflammatory effects observed in mice with DSS-induced IBD after administering the Xianhecao-Huanglian drug pair.

### Xianhecao-Huanglian Drug Pair Promotes Autophagy in Cells Stimulated by LPS via the Signaling Route of JAK2/STAT3

**Fig. 11:**
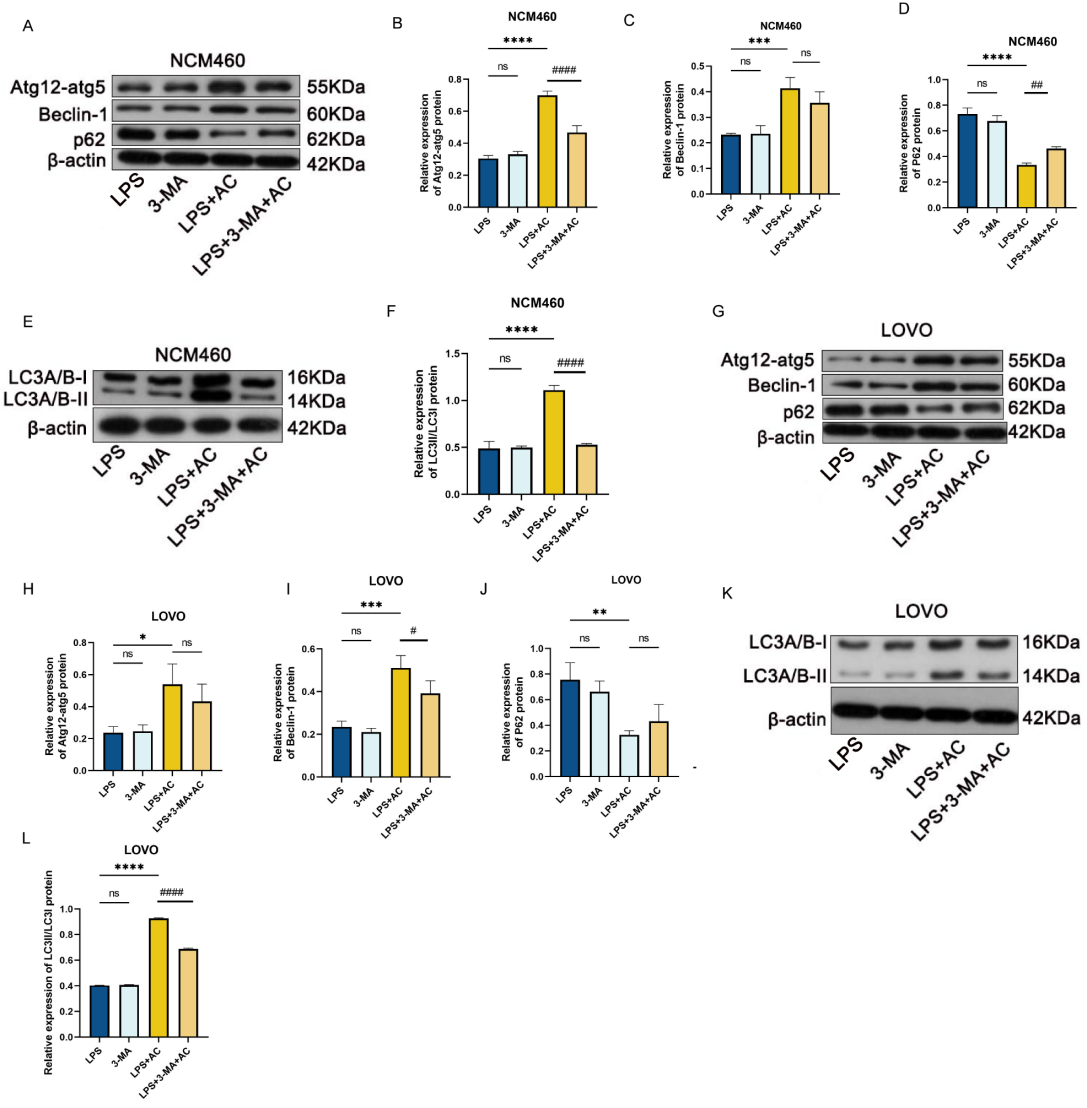
ACPH-DS promotes LPS-induced autophagy in NCM460 and LOVO cells. **A**. Western blot was used to detect the expression of Atg12-atg5, Beclin-1, and p62 proteins in NCM460. The relative protein expression of Atg12-atg5, Beclin-1, and p62 in NCM460 cells of **B-D**. **E**. Western blot was used to detect the expression of LC 3 II/I protein in NCM460. **F**. Relative protein expression of LC 3 II/I .**G**. Western blot was used to detect the expression of Atg12-atg5, Beclin-1, and p62 proteins in LOVO. **H-J**. The relative protein expression of Atg12-atg5, Beclin-1, and p62 in LOVO cells. **K**. Western blot was used to detect the expression of LC 3 II/I protein in LOVO. **L**. Relative protein expression of LC 3 II/I.LPS: Cells treated with 10ug/ml LPS; 3-MA: Treat cells with 5mM of 3-MA; LPS + 3-MA: Pre-treat cells with 5mM 3-MA for 30 min before LPS stimulation; After LPS + 3-MA + AC: 3-MA and ACHP-DS combination pretreatment of cells, LPS was added and co-incubated for 24 h. Data are expressed as the mean ± SD, (*n* = 3). Compared with LPS group, * *p* < 0.05, * * *p* < 0.01, * * * *p* < 0.001, and * * * * *p* < 0.0001; Compared with LPS+AC group,# *p* < ,# # *p* < 0.01, # # # # *p* < 0.0001; ns, not significant

**Fig. 12:**
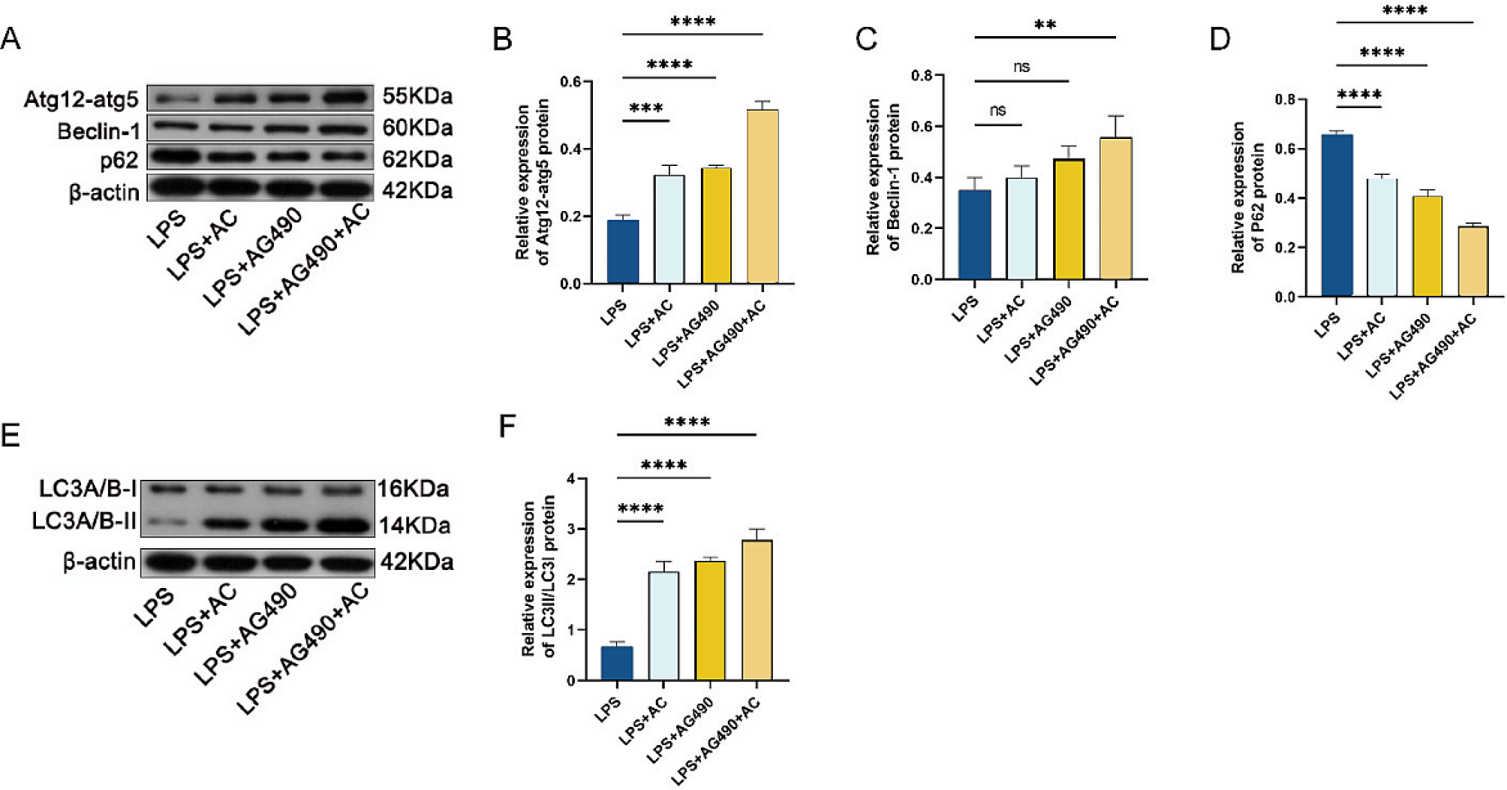
ACPH-DS promotes autophagy by inhibiting the JAK 2/STAT 3 signaling pathway. **A**. Western blot was used to detect the expression of Atg12-atg5, Beclin-1, and p62 proteins in NCM460. **B-D** The relative expression levels of Atg12-atg5, Beclin-1, and p62 proteins. **E**. Western blot was used to detect the expression of LC 3 II/I protein in NCM460. **F**. The relative expression level of LC 3 II/I protein. LPS: Cells treated with 10ug/ml LPS; LPS + AC: 20% ACHP-DS pretreated cells for 2 h, then co-incubated with 10ug/ml LPS for 24 h; LPS+AG490 group: LPS Before stimulation, use AG490 (10 µM) Pre-treat NCM460 and LOVO cells for 1 h; LPS + AG490 + AC: Use AG490 Pre-treat NCM460 and LOVO cells for 1 h, pre-treat NCM460 and LOVO cells with 20% ACHP-DS for 2 h, and add LPS Incubate together for 24 h. Data are expressed as the mean ± SD, (*n* = 3). Compared with LPS group, * * *p* < 0.01, * * * *p* < 0.001, and * * * *f*p* < 0.0001; ns, not significant

Autophagy is closely associated with the development of IBD. In vivo, experiments have demonstrated that the Xianhecao-Huanglian drug pair can regulate autophagy to alleviate DSS-induced acute IBD. To validate this possibility, in vitro experiments were conducted using Western Blot to assess the production of proteins associated with autophagy, including ATG12, Beclin-1, LC3A/B, and p62, in cells. Autophagy was inhibited using 3-MA (5 mM), with LPS + 3-MA serving as the autophagy-negative control group. The results (Fig. 11A-L) revealed that autophagy was enhanced in the LPS-induced cell model when treated with drug-containing serum, as opposed to cells treated with LPS alone. Additionally, it was noted that the 20% drug-containing serum + 3-MA group showed an increase in p62 expression compared to the 20% drug-containing serum group, and a decrease in the expression of autophagy-related proteins ATG12, Beclin-1, and LC3A/B, upon pre-treatment with the autophagy inhibitor 3-MA. This indicates that the autophagy inhibitor 3-MA can impede the autophagy promoted by the drug-containing serum, highlighting the ability of the Xianhecao-Huanglian drug pair to promote autophagy experimentally and in a controlled environment. To further investigate the connection among the Xianhecao-Huanglian drug pair-mediated the route that regulates autophagy as well as the JAK2/STAT3 signaling pathway was inhibited by adding the AG490 inhibitor 30 min in advance. Western blot results (Fig. 12A-F) demonstrated that both the Xianhecao-Huanglian drug pair-containing serum and AG490 could enhance the attenuated autophagy induced by LPS, with the combined effect of both being more significant. These findings illustrate that the Xianhecao-Huanglian drug pair-containing serum promotes autophagy via blocking the signaling route between JAK2 and STAT3.

## Discussion

The concept of “preventing diseases before they occur” holds significant importance in traditional Chinese medicine’s preventive philosophy, emphasizing proactive prevention and disease control, particularly in the context of cancer. Inflammatory conditions often persist during the early stages of mucosal lesions, colorectal adenomas, and the progression to colorectal cancer. Inflammation serves as a pivotal factor in the initial phases of colorectal cancer development, offering an opportune moment for active intervention to reduce colorectal cancer incidence. Traditional Chinese medicine, with its multi-component and multi-target nature, plays a substantial role in intervening in the transition from “inflammation to cancer.” This forms the basis for precision cancer prevention and advances the concept of “preventing diseases before they occur.” Traditional Chinese medicine views the human body as a holistic entity and believes in regulating the balance of yin and yang within the body to enhance self-healing and immunity. This fundamental regulation of the inflammatory response aids in alleviating symptoms and reducing intestinal damage, potentially delaying the progression from inflammatory bowel disease to colorectal cancer.

IBD is classified within the domain of traditional Chinese medicine under categories such as “diarrhea,” “intestinal wind with blood,” “toxin,” and “intestinal obstruction.” “Shen Nong’s Classic of Herbal Medicine” describes Huanglian as primarily employed for addressing “intestinal discomfort, abdominal pain, and diarrhea.” [22] Meanwhile, the “Materia Medica of Southern Yunnan” mentions that Xanhecao is used to manage “bloody and watery diarrhea” [22] Among the many uses for these herbs in TCM, the therapy of gastrointestinal problems is widespread, and contemporary pharmacological investigations have corroborated their potent anti-inflammatory properties [23]. These herbs are now widely applied for the treatment of various inflammation-related disorders. Nevertheless, research on the Xianhecao-Huanglian drug pair for managing IBD is not yet well-established. This study employed an experimental mouse model of IBD caused by DSS and administered the Xianhecao-Huanglian drug pair. Additionally, the anti-inflammatory effects of this treatment were assessed through both in living organisms and in controlled laboratory conditions. The in vivo experiments successfully established an acute IBD mouse model induced by DSS, whereas the in vitro experiments LPS stimulation to generate an inflammatory cell model, illustrating that the Xianhecao-Huanglian drug pair ameliorated pathological damage within the colonic organs of mice with produced IBD and provided significant protection to the colonic mucosal barrier. The potential mechanism may involve autophagy control by means of the JAK2/STAT3 signaling cascade, leading to the reduction of the inflammatory response and the repair of damaged intestinal mucosa, consequently alleviating IBD.

IBD is considered an uncontrollable state of inflammation, characterized by continuous, low-intensity stimulation from pro-inflammatory factors, resulting in persistent inflammation. This chronic inflammation impairs the role of the intestinal tract TJ barrier, thereby promoting the continued production of pro-inflammatory cytokines, thereby establishing a detrimental cycle [26]. Both the development and worsening of IBD are mostly influenced by an inflammatory reaction. Hence, the anti-inflammatory effect of the Xianhecao-Huanglian drug pair was examined and confirmed both in vivo and in vitro. As expected, the Xianhecao-Huanglian drug pair inhibited the secretion of TNF-α, IL-1β, and also IL-6 in the colonic tissues and serum of IBD-afflicted mice, while augmenting IL-10 secretion. This implies that the Xianhecao-Huanglian drug pair may exert its inhibitory effects on IBD by decreasing pro-inflammatory and increasing anti-inflammatory cytokines. Consistent with the in vivo findings, in vitro experiments revealed that serum containing the Xianhecao-Huanglian drug pair reduced the levels of TNF-α, IL-1β, and IL-6 while boosting IL-10 secretion in the LPS-induced IBD inflammatory cell model. Moreover, it was observed that the combination of the Xianhecao-Huanglian drug pair and the JAK2/STAT3 pathway inhibitor AG490 notably decreased inflammatory cytokine levels. This suggests that the Xianhecao-Huanglian drug pair may mitigate inflammation by suppressing the beginning of the JAK2/STAT3 pathway.

An important signaling mechanism that promotes inflammation involves the JAK2/STAT3 pathway. Numerous pieces of literature report continuous STAT3 activation in IBD patients and animal IBD models, underscoring the significant role of STAT3 in intestinal inflammation [27, 28]. JAK2, which belongs to the family of protein-tyrosine kinases, is a precursor of the downstream target gene STAT3. Activated JAK2 can stimulate the phosphorylation of the downstream STAT3 protein, which impacts the development of inflammation [29]. Growing evidence supports that downregulation of JAK2 and STAT3 phosphorylation can mitigate inflammation, as seen in conditions such as cerebral ischemic stroke (IS) injury [30], LPS-induced microglial cell inflammation [29], atopic dermatitis [32], and rheumatoid arthritis (RA) [33]. This study affirms that the Xianhecao-Huanglian drug pair can reverse in both living organisms and laboratory cultures, an abnormal increase of inflammatory cytokines, significantly inhibiting the phosphorylation of JAK2 and STAT3. In vitro experiments revealed that co-treatment with serum containing the Xianhecao-Huanglian drug pair and the JAK2/STAT3 pathway inhibitor AG490 substantially curtailed released inflammatory cytokines and increased their expression in a model of IBD cells. This suggests that the Xianhecao-Huanglian medication combination could reduce inflammation in IBD by blocking the phosphorylation process of JAK2/STAT3.

Autophagy is a cellular process involving the degradation and recycling of cellular materials and organelles, as well as resistance to infection and the removal of intracellular microorganisms [34]. The autophagic process is essential for keeping the mucosal barrier of the intestines in a state of equilibrium and impacts the onset and progression of IBD. Disruption of autophagy is intimately linked to inflammatory disorders [35, 36]. Autophagy is activated in DSS-induced IBD, and this study reveals that the Xianhecao-Huanglian drug pair reverses DSS-induced autophagy inhibition. Animal experiments demonstrated that treatment with the Xianhecao-Huanglian drug pair resulted in increased ratios of ATG12, Beclin-1, and LC3A/B, as well as reduced p62 levels. The same outcomes were consistently observed in in vitro experiments, and it was found that pretreatment with the autophagy inhibitor 3-MA significantly hampered the serum’s ability to promote autophagy. In vitro experiments obtained a consistent conclusion, indicating that pretreatment with 3-MA, which inhibits autophagy, considerably suppressed the drug’s capacity to stimulate autophagy when compared to treatment with the Xianhecao-Huanglian drug pair alone. These findings collectively suggest that the Xianhecao-Huanglian drug pair may alleviate IBD by inducing autophagy. Through in vitro studies, it was observed that the combined use of serum containing the Xianhecao-Huanglian drug pair and the AG490 inhibitor markedly reversed the inhibitory effects on cellular autophagy induced by LPS treatment, as compared to using serum containing the Xianhecao-Huanglian drug pair or AG490 alone. This indicates that the Xianhecao-Huanglian drug pair may promote autophagy by modulating the route for signaling via JAK2/STAT3.

## Conclusion

In summary, in a battery of controlled laboratory and live animal studies, it has been demonstrated that the Xianhecao-Huanglian drug pair exhibits the capability to mitigate and protect the colonic mucosal barrier against DSS-induced acute IBD in mice in LPS-induced NCM460 and LOVO cell models. This protective effect includes the attenuation of inflammation and the preservation of the intestinal mucosa. Regulating the JAK2/STAT3 signaling pathway seems to be the fundamental process, which promotes autophagy as well as reduces phosphorylation of JAK2 along with STAT3. These research findings offer valuable theoretical insights into the clinical management of IBD and the management and avoidance of IBD-related colorectal cancer.

## Data Availability

The original contributions presented in the study are included in the article.
